# Transforming healthcare in Libya – the need for clinical practice guidelines in disease management

**DOI:** 10.1080/20523211.2025.2565419

**Published:** 2025-10-03

**Authors:** Ramadan M. Elkalmi

**Affiliations:** Faculty of Medicine, University of Sebha, Sebha, Libya

**Keywords:** Libya, CPGs, health system reform, health policy, disease management

## Abstract

The healthcare system in Libya faces significant challenges due to political instability, fragmented infrastructure, and inconsistent medical practices. Clinical Practice Guidelines (CPGs) serve as essential tools for standardising care, ensuring evidence-based treatment, and optimising healthcare resources. In Libya, the lack of structured guidelines has contributed to disparities in disease management, affecting patient outcomes and overall healthcare efficiency. This commentary explores the critical need for CPGs in Libya, highlighting their potential to improve healthcare delivery, minimise variability in treatment, and enhance patient safety. While implementation poses challenges, including centralisation, limited research capacity, and resource constraints, integrating CPGs through a phased implementation framework could be a transformative step toward a more resilient and equitable healthcare system. By fostering collaboration among policymakers, healthcare professionals, and international organisations, Libya can lay the foundation for a systematic approach to disease management, ultimately improving the quality of care for its population. Healthcare reform in Libya is urgently needed, and strategic investments in CPG development and dissemination could drive the necessary transformation in Libyan healthcare.

## Introduction

Clinical practice guidelines (CPGs) are systematically developed statements that assist healthcare providers and patients in making decisions about appropriate healthcare for specific clinical circumstances (Rao & Tandon, 2017). In many countries worldwide, CPGs are considered a cornerstone of disease management. They serve as a framework that ensures the delivery of high-quality, evidence-based care, which in turn improves patient outcomes, minimises unnecessary interventions, and optimises healthcare resources (Institute of Medicine (US), 2011). In Libya, a country transitioning through a challenging period of social, political, and economic upheaval, the need for structured healthcare systems, including clinical practice guidelines, has become more pronounced (World Health Organization, 2021). This commentary aims to explore the necessity of CPGs in disease management in Libya and whether this is the right time to begin the transformation towards a more structured healthcare system.

Libya, like many other nations, informally depends on the medical expertise and treatment protocols from developed countries, both in the East and the West. This reliance, coupled with the vast inconsistencies in healthcare practices, subjects patients to significant risks and leads to a decrease in the quality of healthcare services provided in hospitals and health institutes throughout the country. To the best of my knowledge, there are no official or formal instructions regarding using or adaptation of clinical practice guidelines to be used or established in the health setting in the country.

## The necessity of clinical practice guidelines in disease management

Clinical practice guidelines have several advantages, particularly in settings where there is variability in the practice of medicine or limited access to continuous professional development. In Libya, where there is a shortage of specialised healthcare professionals and inconsistent access to medical resources, CPGs can provide an essential framework for disease management. They help standardise the care process and ensure that patients receive the most effective treatment based on the latest scientific evidence (Sevransky et al., 2021). To make this framework actionable, it is crucial to prioritise specific conditions for initial guideline development. Libya’s burden of noncommunicable diseases, such as diabetes mellitus, hypertension, and cardiovascular conditions, continues to grow, requiring standardised chronic care strategies. Simultaneously, infectious diseases, including tuberculosis and viral respiratory infections, present pressing concerns in conflict-affected and underserved regions. Additionally, maternal and neonatal health presents significant challenges, and clear clinical guidelines could substantially improve outcomes across both urban and rural settings. This is particularly important in managing chronic diseases, infectious diseases, and emerging health concerns (Badawi et al., 2019).

One key reason for the necessity of CPGs in Libya is the fragmentation of the healthcare system. The Libyan healthcare infrastructure has faced years of neglect, compounded by the effects of political instability and persistent conflict (El Taguri et al., 2008). As a result, healthcare services are often delivered in an ad hoc and inconsistent manner. CPGs can help mitigate this variability by establishing evidence-based standards for care. For instance, in managing diabetes, cardiovascular diseases, or infectious diseases like tuberculosis, clear and actionable guidelines can ensure that patients across the country receive treatment that adheres to the best clinical practices (Cassidy et al., 2021). This would reduce the risk of misdiagnosis, inappropriate treatment, and suboptimal outcomes (Institute of Medicine, 2015; Institute of Medicine (US), 2011; Singh & Graber, 2015).

Another compelling argument for the necessity of CPGs is the quality assurance they offer. In a setting like Libya, where continuous medical education might not always be accessible, CPGs provide an easy-to-follow resource that helps bridge the knowledge gap and updates clinicians on the most current therapeutic methods. For healthcare professionals in underserved or rural areas, access to updated clinical guidelines can enhance the overall quality of care and reduce the impact of resource limitations (Curran et al., 2006).

## Challenges in implementing clinical practice guidelines in Libya

While the benefits of CPGs are undeniable, there are several challenges to their implementation in Libya. One of the main obstacles is the lack of a centralised, comprehensive healthcare system. Libya has a decentralised healthcare model, which means that healthcare practices can vary significantly between regions, hospitals, and even individual practitioners (Allen et al., 2024). This decentralisation creates challenges in establishing and enforcing standardised guidelines across the country.

Moreover, the political instability that has plagued Libya for over a decade has weakened its healthcare system (Abasse et al., 2021). While there have been efforts to reform the system, persistent conflict and a lack of coordination between the government, healthcare providers, and international organisations have hindered progress. Implementing CPGs would require a stable governance framework, resources for training and dissemination, and strong political will – all of which remain difficult to achieve in the current context (Daw, 2017).

Another barrier is the limited availability of research and evidence-based resources to develop contextually relevant guidelines. Libya faces challenges in conducting clinical research due to insufficient funding, lack of infrastructure, and the absence of a robust research culture in medicine (Elkhammas et al., 2023). CPGs are often based on rigorous scientific research and data, but in a country like Libya, where local evidence is limited, the development of these guidelines may not reflect the specific challenges faced by Libyan patients. Thus, external guidelines that are not tailored to the local context could potentially be ineffective or even harmful if not adapted carefully (Alashek et al., 2011). For CPGs to be successful, they must reflect both the clinical and cultural realities of the Libyan context. This includes consideration of regional healthcare delivery models, sociocultural beliefs about treatment, and logistical constraints such as limited diagnostic capacity. Collaborations with neighbouring countries – such as Tunisia, Egypt, or Algeria – can provide invaluable models for contextual adaptation. These nations share similar post-conflict health challenges and have made strides in guideline localisation and dissemination. Such partnerships could foster regional alignment and capacity-sharing while ensuring that Libyan CPGs remain culturally sensitive and pragmatically grounded.

To enhance regional contextualisation, we have incorporated examples from Iraq and Sudan, two post-conflict countries that have made partial strides in clinical guideline development. Iraq has initiated chronic disease protocols through partnerships with international agencies (Abusaib et al., 2020; Schmid et al., 2022), while Sudan has adapted infectious disease guidelines in response to humanitarian challenges. These cases highlight feasible strategies for guideline adaptation in environments with resource limitations and political fragility (Elamin et al., 2024; Ibrahim et al., 2023). Libya can benefit from similar regional models by engaging in knowledge exchange and tailoring best practices to its healthcare landscape.

## The right time for transformation

Considering the challenges facing Libya’s healthcare system, the question arises: Is this the right time for transformation? On the one hand, the country is still grappling with significant political and economic instability, which could make the task of transforming the healthcare system particularly daunting. On the other hand, the current situation in Libya also presents an opportunity for transformation.

The sustained turmoil in Libya has highlighted the importance of building resilient healthcare systems capable of withstanding crises. In times of conflict or disaster, the ability to deliver high-quality, standardised care is critical to saving lives and preventing long-term health consequences. Clinical practice guidelines can be a vital tool in ensuring that healthcare delivery is consistent, effective, and adaptable in such circumstances (Burgers et al., 2013). With the right investment in infrastructure, training, and collaboration, the implementation of CPGs could help Libya’s healthcare system emerge stronger and more capable of addressing both current and future challenges (Moleman et al., 2022).

Furthermore, there is a growing recognition among Libyan healthcare professionals and policymakers that the country's healthcare system needs modernisation. Although the implementation of clinical practice guidelines would require significant effort, this could also be a catalyst for other much-needed reforms. The process of developing and adopting CPGs could spur improvements in medical education, research, and the infrastructure necessary to deliver evidence-based care. It could also serve as a stepping stone toward the broader transformation of the healthcare system into one that is more organised, efficient, and patient-centered (Beauchemin et al., 2019). Libya’s transition to a more stable and functional healthcare system will depend on the active involvement of both local and international stakeholders. International organisations such as the World Health Organization (WHO) and Médecins Sans Frontières (MSF) have already been providing support to the Libyan healthcare sector, and their experience could be invaluable in the development and implementation of CPGs. Moreover, Libya can learn from other countries that have successfully integrated CPGs into their healthcare systems despite facing similar challenges (Al-Areibi, 2019; Hert & Paula-Garcia, 2024; World Health Organization, 2021).

## Steps toward the successful implementation of clinical practice guidelines

To successfully implement clinical practice guidelines in Libya, several steps need to be taken. First, there must be a concerted effort to establish a national framework for CPG development. This would involve identifying key diseases and conditions that affect the Libyan population and prioritising them in terms of need for standardised guidelines. Collaborating with international bodies, such as the WHO, could help ensure that the guidelines are evidence-based and aligned with global best practices (Alderwick et al., 2021; Joshua et al., 2019; Khalili et al., 2019).

Next, there must be investment in local capacity-building. This includes training healthcare professionals on the importance and use of CPGs, as well as providing sustained support to ensure that guidelines are followed in practice. Local medical institutions and universities can play a central role in educating the next generation of healthcare providers about evidence-based medicine and the importance of clinical guidelines (Celletti et al., 2011).

A third step would be to improve data collection and research within Libya. This could be achieved through collaboration with regional and international research organisations and a focus on developing local clinical data to guide future guidelines. Enhancing Libya’s research infrastructure is crucial for developing clinical practice guidelines that are both locally relevant and practically applicable (Bosch et al., 2007).

A phased implementation strategy is recommended to ensure feasibility and long-term sustainability. Pilot initiatives should begin in stable urban centres such as Tripoli, Misrata, and Benghazi, where comparatively stronger infrastructure allows for structured guideline deployment, workforce training, and systematic feedback collection. These regions offer optimal conditions for stakeholder coordination and digital integration. Insights from pilot programs can then guide a scaled rollout to rural and conflict-affected areas, with appropriate contextual adaptations. To bridge infrastructure disparities, mobile-accessible platforms and digital applications can empower clinicians to retrieve guidelines, participate in virtual training, and document usage patterns – enhancing both reach and consistency in care delivery.

To facilitate the systematic integration of clinical practice guidelines within Libya, the following simplified stepwise framework has been proposed. Table 1 highlights critical milestones – from initial prioritisation through to comprehensive national rollout and monitoring – aligned with the specific demands of Libya’s healthcare infrastructure. This framework is designed to improve transparency, foster effective collaboration among stakeholders, and support the translation of policy into actionable practice. An inclusive, multidisciplinary task force should be established to oversee the development, adaptation, and dissemination of clinical practice guidelines. This body should consist of healthcare professionals, public health experts, policymakers, academic institutions, and representatives from NGOs and civil society. Their collaborative input will ensure that the guidelines are evidence-based, culturally responsive, and logistically feasible. Notably, Libya's medical diaspora presents an untapped reservoir of expertise and international experience. Engaging diaspora professionals in the guideline process can facilitate knowledge exchange, capacity building, and foster global partnerships that support sustainable healthcare reform.

**Table 1.** Key steps to CPG implementation in Libya.

Step	Action	Purpose
1	Prioritise disease areas	Focus initial efforts on high-burden conditions such as diabetes, hypertension, infectious diseases, and maternal/neonatal health.
2	Stakeholder task force formation	Engage clinicians, policymakers, NGOs, and diaspora professionals to guide development.
3	Guideline drafting & adaptation	Use global frameworks (e.g. WHO, MAGICapp) and adapt to Libya’s clinical and cultural context.
4	Pilot implementation in stable regions	Launch in Tripoli, Misrata, and Benghazi to refine processes and gather feedback.
5	Digital integration	Deploy mobile apps and online access for guideline retrieval and virtual training.
6	Nationwide rollout with localisation	Expand with contextual modifications to underserved and conflict-affected areas.
7	Monitoring & evaluation	Track treatment consistency, clinical outcomes, and provider adoption using metrics and feedback tools.

Finally, monitoring and evaluation will be essential in ensuring that the guidelines are effectively integrated into clinical practice. A robust system for tracking outcomes, patient satisfaction, and healthcare quality will help identify areas for improvement and make adjustments to the guidelines as needed (Hughes, 2008). Monitoring and evaluation will be essential for assessing the impact of clinical practice guidelines. Key metrics may include reductions in treatment variability across regions, improvements in disease management outcomes (e.g. glycemic control for diabetes, reduced complications from infections), and adoption rates among healthcare providers. The use of digital tools can support data collection and trend analysis, while patient satisfaction surveys and training assessments will further inform the quality and reach of guideline implementation.

## Conclusion

Clinical Practice Guidelines (CPGs) are essential for improving disease management and catalyzing health system reform in Libya. Despite prolonged instability, the need for structured, evidence-based care has never been more urgent. CPGs can reduce treatment variability, improve outcomes, and support equitable healthcare across diverse regions. Implementing them requires investment in training, local research, digital tools, and collaboration with national stakeholders, international organisations, and diaspora professionals. These actions will lay the foundation for a resilient, efficient, and patient-centered health system. The time to begin this transformation is now.
